# A Multimodal Quality Improvement Initiative to Reduce Non-ICU Telemetry Overutilization

**DOI:** 10.1016/j.jaccas.2026.109232

**Published:** 2026-09-16

**Authors:** Taysir Al Janabi, Jack Anderson, Moataz Ali, Karampal Mand, Deepika Jain

**Affiliations:** a Department of Internal Medicine, WellSpan York Hospital, York, Pennsylvania, USA; b Department of Internal Medicine, Drexel University College of Medicine, Philadelphia, Pennsylvania, USA; c Department of Internal Medicine, Ivano-Frankivsk National Medical University, Ivano-Frankivsk, Ukraine

**Keywords:** alarm fatigue, hospital medicine, quality improvement, telemetry, value-based care, workflow optimization

## Abstract

**Background:**

Continuous cardiac telemetry is frequently overutilized in hospitalized non–intensive care unit patients beyond guideline-supported indications.

**Project Rationale:**

Excessive telemetry use contributes to alarm fatigue, resource strain, increased health care costs, and unnecessary patient monitoring.

**Project Summary:**

A quasi-experimental before-and-after study was conducted at a community hospital. A multidisciplinary team implemented a multimodal intervention that included 1) changing telemetry order expiration to noon on hospital day 2, 2) conducting targeted resident education regarding American Heart Association telemetry indications, 3) implementation of a visible telemetry indicator within the electronic medical record, and 4) distribution of pocket reference cards outlining telemetry indications and discontinuation criteria. Sixteen weeks of preintervention data were compared with 16 weeks of postintervention data. The primary outcome was the reduction in telemetry orders per resident, analyzed using the Wilcoxon signed-rank test.

**Take-Home Messages:**

A simple workflow modification supported by focused resident education significantly reduced non–intensive care unit telemetry ordering. This intervention was low cost, sustainable, and easily reproducible.

## Visual Summary

**Figure:**
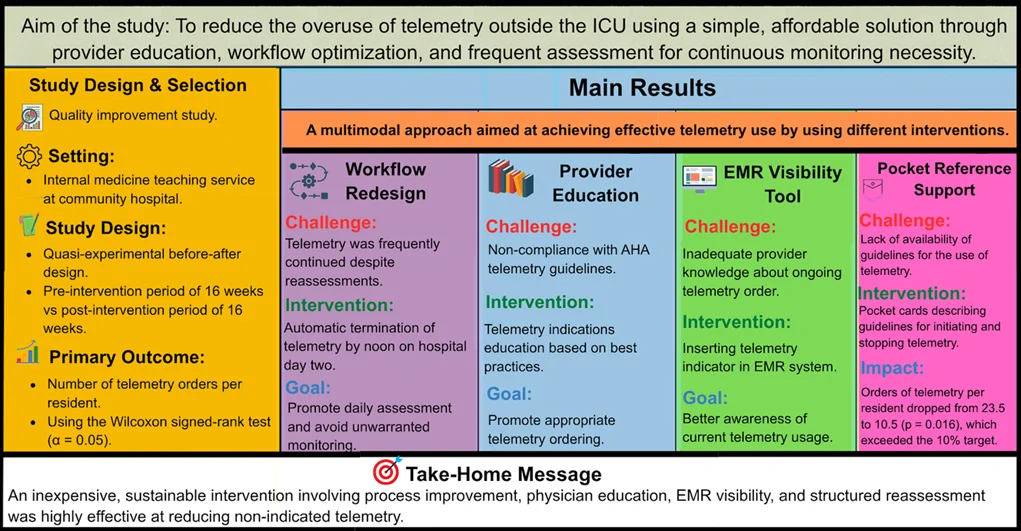

**Visual Summary:**
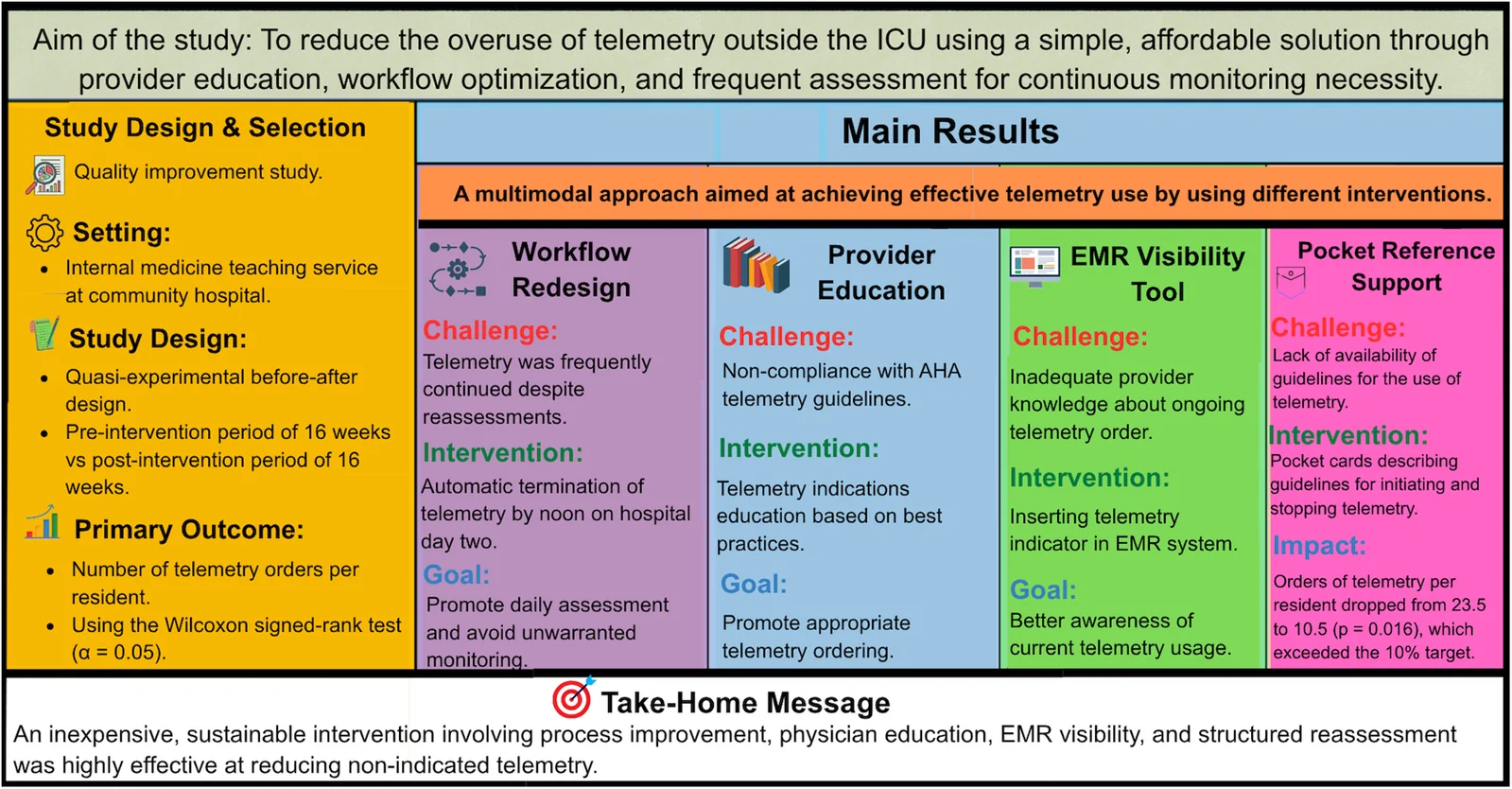
Multimodal Interventions Significantly Reduced Non-ICU Telemetry Utilization AHA = American Heart Association; EMR = electronic medical record; ICU = intensive care unit.

Continuous cardiac telemetry remains an important component of inpatient monitoring for patients at risk of clinically significant arrhythmias and cardiac instability. Current American Heart Association (AHA) guidelines provide evidence-based recommendations regarding appropriate telemetry utilization and emphasize the importance of selective patient monitoring.^1^ Despite these recommendations, telemetry overutilization remains common, particularly among non–intensive care unit (ICU) patients.^2^, ^3^, ^4^

**Take-Home Messages**

• Inappropriate telemetry utilization remains common among non-ICU hospitalized patients.
• A multimodal telemetry optimization intervention can significantly reduce unnecessary telemetry ordering.

Several studies have shown that telemetry is frequently continued in patients without ongoing clinical indications, contributing to unnecessary health care costs, alarm fatigue, inefficient resource utilization, and workflow interruptions without clear improvement in clinical outcomes.^2^^,^^4^, ^5^, ^6^, ^7^ Excessive alarm burden also may contribute to staff desensitization and decreased responsiveness to clinically significant events, raising additional patient safety concerns.^6^^,^^8^ Prior investigations also have demonstrated variability in telemetry utilization practices across different clinical settings, further highlighting opportunities for workflow standardization and resource optimization.^9^

As health care systems continue to prioritize value-based care and resource stewardship, reducing inappropriate telemetry utilization has become an increasingly important target for quality improvement initiatives. Previous interventions aimed at reducing telemetry overutilization have focused on resident education, guideline-based ordering systems, clinical decision support tools, and structured reassessment workflows.^3^^,^^10^ Several studies have suggested that these interventions are most effective when integrated directly into routine clinical workflow rather than relying solely on passive educational efforts or restrictive ordering practices.^3^^,^^10^

Before January 2021, each hospital floor in our institution had a telemetry unit staffed with monitoring technicians who reported significant events to the care team. However, these technicians were also tasked with some administrative duties. To enhance efficiency, minimize distraction, and improve patient safety, central telemetry monitoring was established in January 2021. This central telemetry monitoring team monitored telemetry across all hospital floors from a single unit. The implementation of centralized telemetry monitoring was associated with a substantial increase in telemetry utilization among patients admitted to the internal medicine teaching service. Internal review demonstrated that more than 88% of hospitalized patients on the teaching service were placed on telemetry, suggesting significant use beyond guideline-supported indications. Diagnoses of patients placed on telemetry varied and included some noncardiac diagnoses. Some examples of telemetry use on noncardiac patients are HIV infection, diabetic foot infection, and sepsis. This increase contributed to operational strain, alarm burden, and concerns regarding unnecessary monitoring among low-risk patients.

In response, we developed a multidisciplinary quality improvement (QI) initiative aimed at reducing inappropriate telemetry utilization through workflow redesign, resident education, and structured telemetry reassessment. The primary objective of this initiative was to achieve at least a 10% reduction in telemetry utilization while preserving high-quality inpatient care.

## Case Summary: Prompting the Project Launch

Continuous telemetry monitoring was frequently initiated for non-ICU patients without clear evidence-based indications. Following centralized telemetry implementation at our hospital, telemetry utilization increased substantially among patients admitted to the internal medicine teaching service.

At baseline, more than 88% of patients admitted to the teaching service were placed on telemetry. This increase contributed to alarm fatigue, operational inefficiencies, increased equipment utilization, and additional health care costs. The project team identified an opportunity to improve value-based care through implementation of a standardized, guideline-informed telemetry optimization strategy.

## Project Rationale

Overutilization of telemetry has important implications for both patient care and health care systems. Excessive telemetry use may contribute to alarm fatigue among health care staff, increase unnecessary interruptions in workflow, and strain telemetry resources without meaningful clinical benefit.^2^^,^^6^

The AHA provides clear recommendations regarding appropriate inpatient telemetry monitoring; however, adherence to these guidelines remains inconsistent in many clinical settings.^1^ Prior studies have shown that physician education, workflow redesign, and structured reassessment processes may reduce inappropriate telemetry utilization.^3^^,^^10^

This project aimed to promote evidence-based telemetry ordering practices while improving workflow efficiency and reducing unnecessary monitoring among low-risk hospitalized patients.

## Project Description

A multidisciplinary team conducted a quasi-experimental before-and-after study within the internal medicine teaching service at a community hospital.

The intervention consisted of 4 major components:

1. Telemetry order expiration was changed to noon on hospital day 2 to prompt reassessment during daily rounds.
2. Targeted resident education sessions were conducted regarding AHA telemetry indications and evidence-based telemetry utilization.
3. A visible telemetry indicator was incorporated into the electronic medical record to improve awareness of active telemetry orders.
4. Pocket reference cards outlining telemetry indications and discontinuation criteria were distributed to residents.

Telemetry order data were extracted from the electronic medical record. Sixteen weeks of preintervention data (May 24–September 11, 2024) were compared with 16 weeks of postintervention data (October 9, 2024–January 29, 2025). Telemetry use was measured and analyzed at the individual resident level rather than as an aggregate measure of total telemetry use. Comparing each resident's pre- and postintervention telemetry utilization was intentionally chosen because it allowed residents to serve as their own controls. This approach enabled the project team to account for resident-specific factors that may affect telemetry use and provided a more rigorous assessment of changes associated with the intervention than an analysis of overall telemetry utilization alone. The primary outcome was the number of telemetry orders per resident. Statistical analysis was performed using the Wilcoxon signed-rank test with an alpha level of 0.05.

### Project Deliverables

Project deliverables included revised telemetry order workflows, educational sessions on telemetry indications, electronic medical record–based telemetry visibility tools, and pocket reference cards summarizing telemetry initiation and discontinuation criteria (Figure 1). These interventions established a standardized reassessment process and reinforced evidence-based telemetry utilization practices among residents.

**Figure 1:**
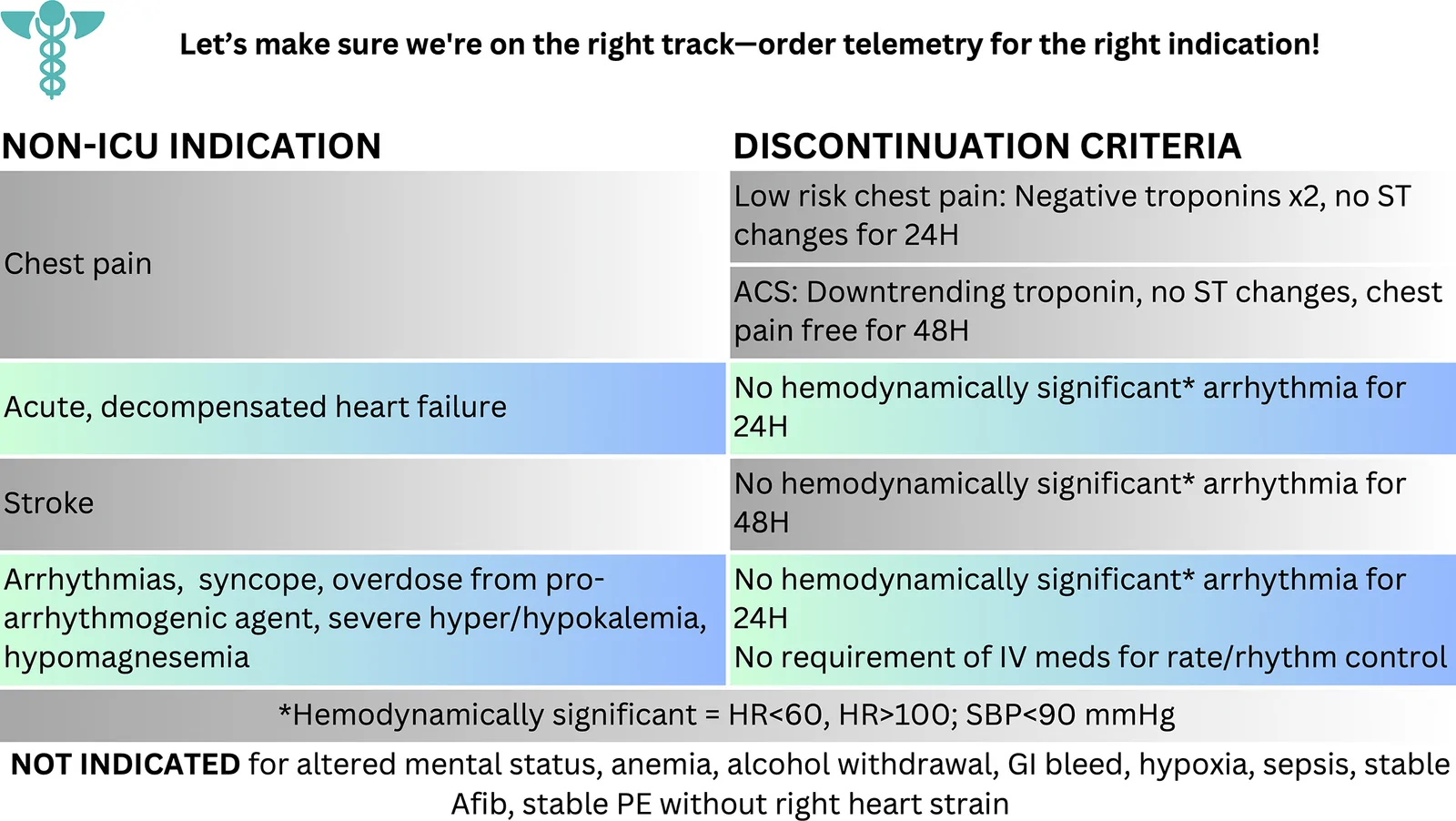
Pocket Reference Card Pocket reference tool developed to support evidence-based telemetry ordering and discontinuation among non-ICU patients as part of the multimodal telemetry optimization intervention. ACS = acute coronary syndrome; GI = gastrointestinal; HR = heart rate; ICU = intensive care unit; PE = pulmonary embolism; SBP = systolic blood pressure.

## Project Outcome, Impact, and Future Directions

The intervention resulted in a substantial reduction in telemetry ordering among residents on the internal medicine teaching service. The total number of residents in our program was 36. Six residents were excluded because there were no orders under their names. Subsequently, only 30 residents were included in paired analysis. Median telemetry orders per resident decreased from 23.5 (IQR: 13.25-39.5) during the preintervention period to 10.5 (IQR: 6.25-17.5) during the postintervention period (*z* = −2.418, *P* = 0.016). Although telemetry duration per order did not significantly change, the overall reduction in telemetry utilization exceeded the project's original target of a 10% reduction (Figure 2). Figure 3 shows the change and variability over time in per-resident telemetry orders per month. Beyond reductions in telemetry use, the intervention improved resident awareness regarding evidence-based telemetry indications and promoted structured reassessment of ongoing monitoring needs.

**Figure 2:**
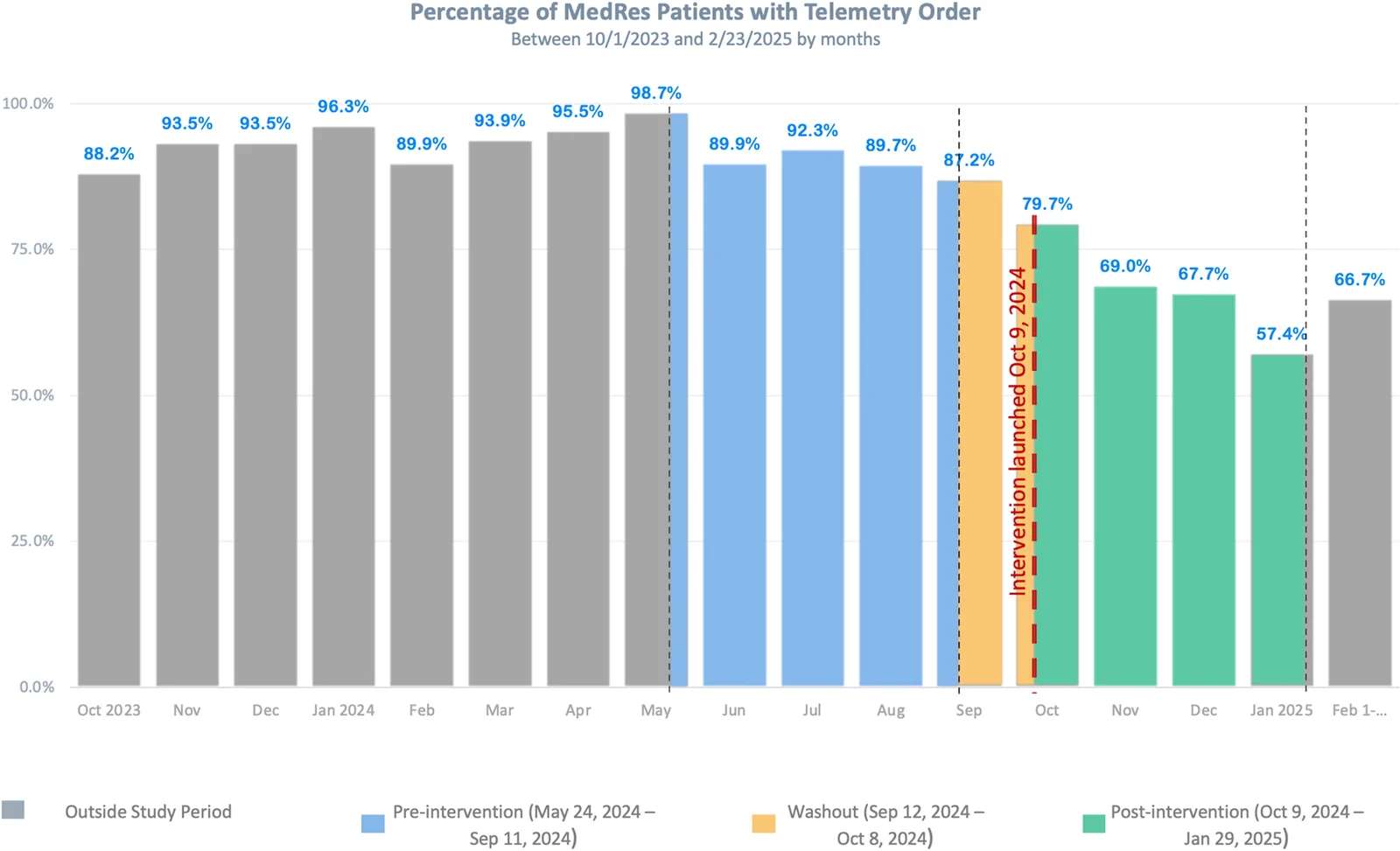
The Timeline of the Implementation and Impact of Telemetry Intervention Monthly percentages of internal medicine teaching service patients with active telemetry orders before-and-after implementation of a telemetry optimization intervention.

**Figure 3:**
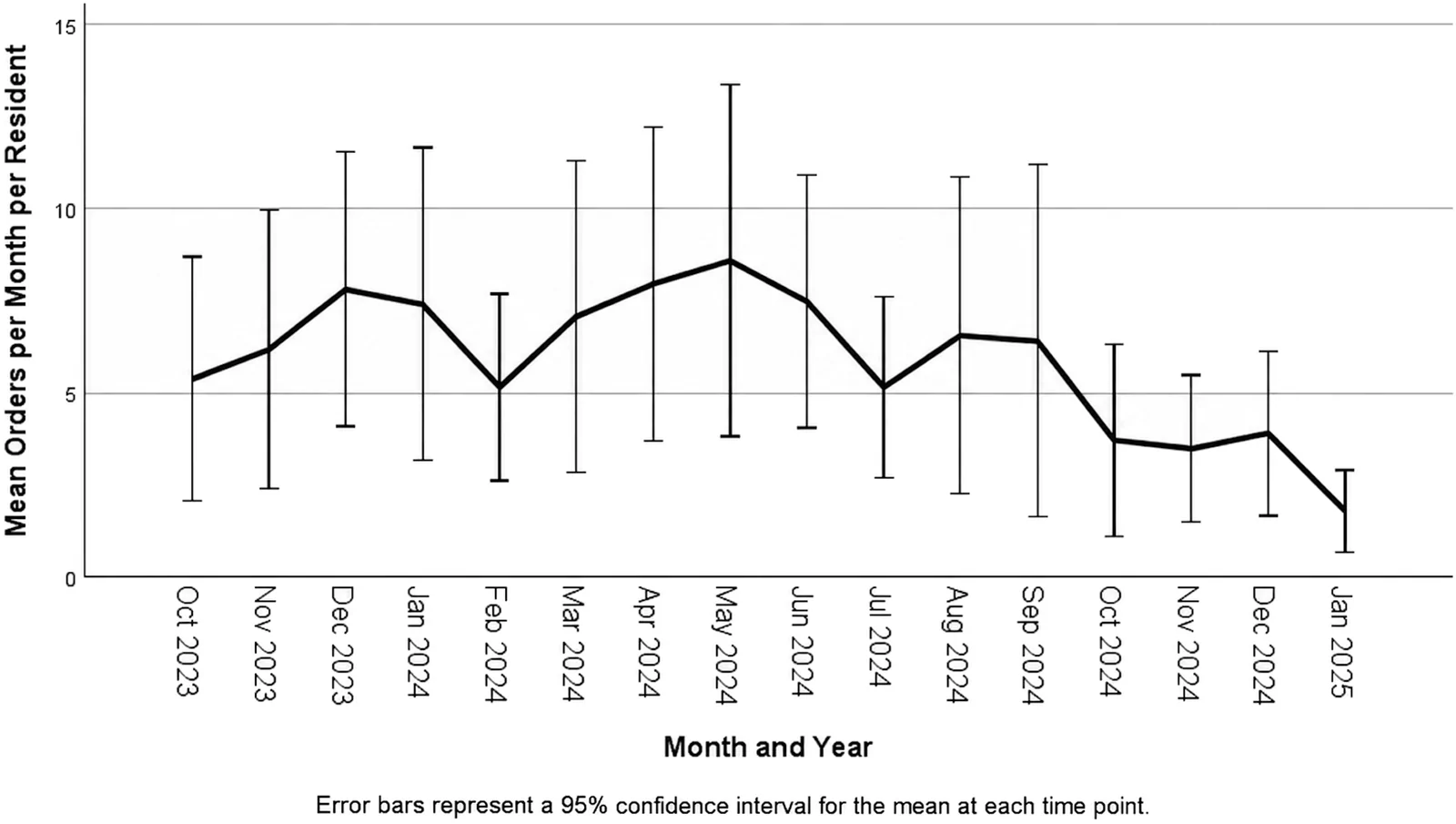
The Mean Telemetry Orders per Month per Resident Before the Study Period, in the Pre-intervention period, Washout Period, and Post-intervention Period A line graph showing the average number of telemetry orders per resident per month, covering October 2023 to January 2025. The chart shows that since the launch date of October 9, 2024, there has been a decrease in the average telemetry order per resident per month, coupled with decreased variance.

Future directions include sustaining reductions in telemetry overutilization, integrating telemetry reassessment into routine inpatient workflow, and expanding the intervention to additional hospital services.

## Discussion

This QI initiative demonstrated that a simple, guideline-informed intervention was associated with a significant reduction in non-ICU telemetry utilization on an internal medicine teaching service. Following implementation of workflow modification, resident education, and structured reassessment processes, telemetry orders per resident decreased substantially and exceeded the project's predefined reduction target.

A key contributor to this improvement was likely the integration of telemetry reassessment into routine clinical workflow. Automatic expiration of telemetry orders on hospital day 2 created a standardized reassessment point during daily rounds and reduced passive continuation of monitoring. In addition, focused education regarding AHA telemetry indications and the use of visible telemetry indicators within the electronic medical record increased resident awareness of active telemetry orders and reinforced evidence-based ordering practices.^1^^,^^3^^,^^10^ Previous studies evaluating telemetry reduction initiatives have similarly demonstrated that resident engagement and workflow-based reassessment strategies are associated with more sustainable reductions in telemetry utilization.^3^^,^^10^ However, our QI project did not show a change in telemetry duration, which might be attributable to the fact that, for individuals in whom telemetry was indicated, their orders were renewed beyond AHA indications.

These findings are clinically relevant given the growing emphasis on value-based inpatient care. Telemetry overutilization has been associated with alarm fatigue, unnecessary resource utilization, increased health care costs, and workflow inefficiencies without clear improvement in patient outcomes.^2^^,^^4^, ^5^, ^6^ Excessive nonactionable alarms may contribute to staff desensitization and delayed responses to clinically significant alarms, particularly in high-volume inpatient environments.^6^^,^^8^ By reducing unnecessary telemetry utilization, our intervention may help improve resource allocation while maintaining appropriate patient monitoring.

Our findings are consistent with prior studies demonstrating that educational interventions, guideline-based workflows, and electronic monitoring tools can reduce inappropriate telemetry utilization.^3^^,^^10^ Previous investigations have also suggested that workflow redesign strategies integrated directly into routine clinical practice may improve resident adherence and sustainability of telemetry reduction efforts over time.^10^ Unlike restrictive ordering systems, our intervention emphasized resident awareness and clinical reassessment, which may improve long-term acceptance among frontline clinicians.

An additional strength of this project was its practicality. The intervention required minimal financial investment and relied primarily on workflow redesign and resident education rather than major infrastructure changes or additional staffing. The use of electronic medical record–based visibility tools also allowed telemetry reassessment to be incorporated directly into existing inpatient workflow. As a result, this approach may be reproducible across other residency-based teaching services and hospital systems facing similar challenges with telemetry overutilization.

### Study limitations

This study was conducted at a single community hospital and may not be generalizable to institutions with different patient populations or telemetry workflows. In addition, the study evaluated telemetry ordering behavior without assessing downstream clinical outcomes such as arrhythmia detection, rapid response events, or mortality. Prior studies have demonstrated variability in telemetry practices across institutions, which may influence the reproducibility of intervention outcomes.^4^^,^^9^ Finally, because this was a quasi-experimental QI initiative, the relative contribution of each intervention component to the observed reduction in telemetry utilization could not be independently determined.

Despite these limitations, this project demonstrates that relatively simple workflow-based interventions can meaningfully reduce inappropriate telemetry utilization while supporting efficient and value-based inpatient care.

## Conclusions

A structured, guideline-informed intervention significantly reduced non-ICU telemetry utilization among patients admitted to the internal medicine teaching service. Workflow redesign, resident education, and structured reassessment processes were associated with meaningful reductions in telemetry ordering while preserving high-quality patient care. This low-cost and sustainable intervention may serve as a reproducible framework for other institutions seeking to optimize telemetry utilization and promote value-based inpatient care.

## Funding Support and Author Disclosures

The authors have reported that they have no relationships relevant to the contents of this paper to disclose.
